# Supplementary material for: Daptomycin Population Pharmacokinetics in Patients Affected by Severe Gram-Positive Infections: An Update
Source: Antibiotics (Basel). 2022 Jul 7;11(7):914. doi: 10.3390/antibiotics11070914 (PMC9311615; doi:10.3390/antibiotics11070914)
Supplement: Supplementary file 1 [file antibiotics-11-00914-s001.zip › antibiotics-1761167-supplementary.pdf]

Daptomycin population pharmacokinetics in patients affected by severe Gram-positive infections: an update

Balice G.; Passino C.; Bongiorni M.G.; Segreti L.; Russo A.; Lastella M.; Luci G.; Falcone M.; Di Paolo A.

Supplementary material

Table S1. Dose-stratified cumulative fraction of response for MRSA (AUC/MIC 666). Data for the 12 mg/kg daily doses were uninformative and thus omitted.

Daily dose	MIC range (mg/L)								CFR
(mg/kg)	0.25	0.5	1	2	4	8	16	32	
4	100	97.55	22.69	1.11	0.11	0.04	0	0	88.83
6	100	100	66.72	4.42	0.21	0.03	0.03	0	95.77
8	100	100	95.63	19.02	0.96	0.19	0	0	99.44
10	100	100	99.27	27.62	0.94	0.10	0.05	0	99.91

Abbreviations: CFR, cumulative fraction of response; MIC; minimum inhibitory concentration.

Table S2. Dose-stratified cumulative fraction of response for *S, pneumoniae* (AUC/MIC 438). Data for the 12 mg/kg daily doses were uninformative and thus omitted.

Daily dose	MIC range (mg/L)								CFR
(mg/kg)	0.25	0.5	1	2	4	8	16	32	
4	100	99.96	77.46	6.79	0.37	0.07	0.04	0	99.92
6	100	100	98.90	27.89	1.15	0.11	0.03	0	99.99
8	100	100	100	86.35	8.23	0.16	0.10	0	100
10	100	100	100	99.04	31.36	1.48	0.19	0	99.99

Abbreviations: CFR, cumulative fraction of response; MIC; minimum inhibitory concentration.

Table S3. Dose-stratified cumulative fraction of response for *E. faecium* (AUC/MIC 294,95). Data for the 12 mg/kg daily doses were uninformative and thus omitted.

Daily dose	MIC range (mg/L)							CFR
(mg/kg)	0.5	1	2	4	8	16	32	
4	100	99.41	36.37	1.78	0.19	0.04	0.04	33.81
6	100	100	83.46	7.85	0.34	0.03	0.03	68.01
8	100	100	99.04	31.36	1.48	0.19	0	83.74
10	100	100	99.95	42.31	1.93	0.16	0.10	86.64

Abbreviations: CFR, cumulative fraction of response; MIC; minimum inhibitory concentration.
